# Training approaches for the dissemination of clinical guidelines for NSSI: a quasi-experimental trial

**DOI:** 10.1186/s13034-024-00789-x

**Published:** 2024-08-10

**Authors:** Elisa Koenig, Ulrike Hoffmann, Jörg M. Fegert, Ferdinand Keller, Maurizio Sicorello, Jennifer Spohrs, Laura Kraus, Sandra Nickel, Christian Schmahl, Birgit Abler, Tina In-Albon, Julian Koenig, Dennis Ougrin, Michael Kaess, Paul L. Plener, the Star-Consortium, Elisa Sittenberger, Lisa Schischke, Alina Geprägs, Isabell Liebhart, Andreas Witt, Cedric Sachser, Rebecca Brown, Vera Münch, Elisa König, Inga Niedtfeld, Jenny Zähringer, Hasan-Hüseyin Isik, Michael Koelch, Olaf Reis, Anna Michelsen, Andreas G. Chiocchetti, Silvia Lindlar, Regina Waltes, Markus Mössner, Patrice van der Venne, Saskia Höper, Elisa Flach, Alexandra Edinger, Stephanie Bauer, Margarete Mattern, Sabine Herpertz, Ulrich Ebner, Philip S. Santangelo

**Affiliations:** 1 Department of Child and Adolescent Psychiatry/Psychotherapy, Ulm University Hospital, Ulm, Germany; 2 German Center of Mental Health (DZPG), Partner site Ulm, Germany; 3 Department of Psychosomatic Medicine and Psychotherapy, Central Institute of Mental Health Mannheim, Medical Faculty Mannheim, Heidelberg University, Mannheim, Germany; 4 Clinical Child and Adolescent Psychology and Psychotherapy, University of Kaiserslautern-Landau, Landau, Germany; 5 German Center of Mental Health (DZPG), Partner site Mannheim, Germany; 6 Department of Psychiatry and Psychotherapy III, Ulm University, Ulm, Germany; 7 Department of Child and Adolescent Psychiatry, Psychosomatics and Psychotherapy, Faculty of Medicine and University Hospital Cologne, University of Cologne, Cologne, Germany; 8 Youth Resilience Unit, WHO Collaborating Centre for Mental Health Services Development, Centre for Psychiatry and Mental Health, Wolfson Institute of Population Health, Queen Mary University of London, London, UK; 9 Department of Child and Adolescent Psychiatry, Centre for Psychosocial Medicine, Heidelberg University, Heidelberg, Germany; 10 University Hospital of Child and Adolescent Psychiatry and Psychotherapy, University of Bern, Bern, Switzerland; 11 Department of Child and Adolescent Psychiatry, Medical University of Vienna, Vienna, Austria

**Keywords:** Training, Guidelines, NSSI, Dissemination, E-learning, Blended-learning, Printed educational material

## Abstract

**Background:**

Non-suicidal self-injury (NSSI) is of high clinical relevance due to its high prevalence and negative long-term implications. In 2016, the German consensus-based clinical guidelines for diagnostic, assessment and treatment of NSSI in childhood and adolescence were published. However, research indicates that clinical guidelines are often poorly implemented in clinical practice. One crucial part of this process is the training of healthcare professionals to transfer knowledge and capacities to bring guideline recommendations into clinical practice.

**Methods:**

The effect of three different dissemination strategies (printed educational material, e-learning, and blended-learning) on the NSSI guidelines´ recommendations was examined among 671 physicians and psychotherapists via an online-survey. The quasi-experimental study included three measurement points (before the training, after the training, 3-month follow-up) and mixed effects models were used to test for changes in knowledge, competences and attitudes toward NSSI and treatment. Moreover, the transfer of gained competences to practical work and user satisfaction were reviewed.

**Results:**

With all three training formats, the intended changes of the outcome variables could be observed. Hereby, the printed educational material condition showed the lowest improvement in the scores for the ‘negative attitudes toward NSSI and those who self-injure’. The training effect remained stable throughout the follow-up measurement. The highest application rate of acquired intervention techniques in clinical practice was reported for the blended-learning condition. For all three training strategies, user satisfaction was high and evaluation of training quality was positive, with printed educational material receiving the lowest and blended-learning the highest evaluations.

**Conclusions:**

In summary, all three training formats were perceived to be of high quality and seem to be suited to cover the needs of a heterogeneous group of physicians and psychotherapists. The choice of training method could be driven by considering which training goals are desired to be achieved and by the benefit-cost ratio allowing for tailored training approaches.

**Supplementary Information:**

The online version contains supplementary material available at 10.1186/s13034-024-00789-x.

## Background

Non-suicidal self-injury is defined as deliberate, self-directed damage of body tissue without suicidal intent and for purposes not socially or culturally sanctioned [1]. Studies found a lifetime prevalence of 17–35% in adolescents for at least one incident of NSSI [2–4]. The DSM-5 proposed diagnostic criteria for NSSI and included it as a condition for further study. The criteria include repetitive nature of the self-injurious behaviour with at least 5 incidents within the last year as well as functional, motivational and emotional aspects of NSSI [5]. Applying these DSM-5 criteria for NSSI, rates among child and adolescent community samples range from 1.5 to 6.7% [6]. Even higher prevalence rates are found in inpatient clinics [7] and in youth welfare [8]. NSSI peaks around the age of 15 and decreases in late adolescence [9, 10] but bears long-term risks, including increased rates of suicide attempts and suicides [11, 12]. Considering the high prevalence of NSSI, it is very likely that a high number of (mental) healthcare professionals get in contact with patients who self-injure at some point during their work including diagnosing, managing, transferring and/or treating NSSI. In conclusion, NSSI is of high clinical relevance due to its high prevalence and long-term implications.

There have been guidelines on the broader concept of self-harm (defined as any intentional self-poisoning or self-injury, irrespective of suicidal intent), such as the guidelines of the National Institute for Health and Care Excellence (NICE) [13–15]; however, it was not until 2016 that the German consensus-based clinical guidelines for diagnostic assessment and ltreatment of NSSI in childhood and adolescence were published as the first internationally published guidelines on NSSI specifically [16, 17]. These guidelines aim at optimizing diagnostic processes and treatment of NSSI, improving care for patients with NSSI [18]. Recent meta-analyses imply that patients treated with guideline-adherent treatments improve to a greater degree and more quickly than patients treated with treatment-as-usual [19] and that guideline implementation strategies may influence patient outcomes positively [19, 20]. However, research indicates that evidence-based clinical guidelines are poorly implemented in clinical practice and require active action for implementation [20–23]; this also applies to mental health guidelines [24–26] and the NICE guideline on self-harm [27]. To overcome this gap, efforts must be made to disseminate evidence-based knowledge into clinical practice. An important prerequisite for guidelines to enfold their positive impact is for professionals to have the necessary knowledge and capacities for evidence-based procedures [19]. Thus, continuous education plays a crucial role in offering healthcare professionals the information they need to deliver patient care according to guideline recommendations [21, 28]. However, studies suggest that there is a lack of training of (mental) health care professionals for self-harm [27, 29–32].

E-learning has become increasingly popular in offering education for (mental) health professionals on evidence-based treatment recommendations, especially during the COVID-19 pandemic [33]. E-learning has the advantage of providing low-threshold, easy and flexible access to training programs and has been proven to be effective in (continuous) education for healthcare professions, being at least as effective as traditional learning approaches [34–39]. The shortcomings of e-learning include the lack of interpersonal communication/exchange and challenges in offering direct practical application of skills including individualized feedback [40–42]. Blended learning, as the combination of e-learning and face-to-face learning, can overcome these shortcomings and combine the advantages of both e-learning and face-to-face learning. According to various meta-analyses, blended learning is at least as effective as traditional learning [43–46] and e-learning [45] in health education. A more ‘traditional’ way of disseminating knowledge is printed educational materials (PEMs). A recent meta-analysis suggested that, when used alone and compared to no intervention, PEMs may slightly improve healthcare professionals’ practice outcomes. However, the effectiveness of PEMs compared to other interventions or of PEMs as part of a multifaceted intervention is uncertain [47].

To date, no studies have provided insights into the effect of dissemination strategies on NSSI guidelines. However, continuous education programs for professionals dealing with the prevention and/or intervention of suicidality have been explored in several studies. One found improved self-perceived knowledge and confidence among staff of psychiatric departments who received a blended-learning train-the-trainer programme about suicide guidelines in contrast to professionals who were exposed only to traditional guideline dissemination (e.g., internet, newsletter, books, publications and congresses) [48]. A blended-learning approach including role-play training for mental health professionals on the assessment and management of suicide risk was also found to improve clinical skills such as perceived behavioural control or the development of a treatment plan [49]. Another study revealed higher levels of self-evaluated knowledge about suicide and greater confidence in having a conversation about suicidal behaviour among undergraduate psychology students receiving an e-learning module compared to a wait list control group [50]. Ghoncheh and colleagues [51] explored suicide prevention e-learning-modules in a wait list control group design among 190 gatekeepers. The results demonstrated that the perceived and actual knowledge and perceived self-confidence of gatekeepers in the experimental group improved significantly compared to those in the wait list control group. These studies show the benefit of blended-learning [48, 49] and e-learning [50, 51] in training health professionals in dealing with suicidality, but they do not directly compare those dissemination strategies. In their meta-analysis of the effectiveness of blended-learning in health professions, Liu and colleagues [45] also concluded that there are only a few studies comparing blended-learning with e-learning. Considering the evidence available about training professionals in evidence-based interventions for mental health in general, there is mounting evidence demonstrating the need for dissemination and training strategies taking an active approach beyond the provision of manuals or self-study [52, 53]. For example, a dissemination trial testing three strategies (manual only, review of the manual plus access to a training Web site, or review of the manual plus a didactic seminar followed by supervised casework) for training 78 community-based clinicians in cognitive-behavioural therapy found that clinicians’ ability was significantly greater for those who participated in the seminar plus supervision condition than for those assigned to the manual only condition, with intermediate ratings for the Web condition [54]. Likewise, meta-analyses point to the potential benefits of simulation-based education, such as role-play in suicidal crisis intervention – whether, for example, as the sole form of intervention or as part of a blended-learning format – in relation to improving attitudes, knowledge, skills and behaviours of health professionals [55–57]. To summarize, the current body of research first indicates the potential of e-learning and blended-learning in the dissemination of evidence-based knowledge and skills and second suggests that more active training approaches, such as role-plays, are more likely to enhance health professionals´ competences. However, to our knowledge, no studies have directly compared different dissemination interventions for evidence-based guidelines, especially regarding NSSI. The aim of the current study was therefore to examine the effect of three different dissemination strategies of the German NSSI guidelines among (mental) healthcare professionals (printed educational material, e-learning, blended-learning) on the acquisition of knowledge, competences, positive attitudes, transfer to practical work and acceptance of the respective dissemination strategies. This selection of training modalities is intended to build on and expand the existing research literature by directly comparing current and promising training approaches (e-learning and blended-learning) while including printed educational material as a widely used, low-threshold and easy-to-create training strategy in the comparison. Based on previous studies, we hypothesized that printed educational material would result in the lowest improvement in all assessed professionals´ outcomes (H1) because it has the lowest interactive approach among the three training modalities. As the blended-learning- and the e-learning-conditions differed only in the presentation of the course module on the specific intervention ‘Therapeutic Assessment’ (see description below), we assumed that blended-learning only performs better than e-learning in the uptake on that intervention into practical work, but that there are no differences between the two conditions in the remaining variables (H2). The study was part of the cooperative project STAR (Self-injury: Treatment, Assessment, Recovery), funded by the German Federal Ministry of Education and Research. The results and procedures are reported in accordance with the CONSORT-SPI 2018 [58, 59].

## Methods

### Design and procedure

Three different training strategies (printed educational material (PEM), e-learning (EL), and blended-learning (BL)) were developed and evaluated with a quasi-experimental pretest-post test design among physicians and psychotherapists.

For recruitment, professional associations and chambers of psychotherapists and physicians were contacted to distribute information about the project by their mailing lists. Additionally, the project was promoted at professional conferences, via mailing lists and social media channels. As the trainings started at four time points during the project phase (see below), recruitment took place between 08/2018 and 08/2020.

Persons interested in the project and the study could register on the project homepage. Eligible persons were included in the study. The study inclusion criteria comprised residence in Germany and being graduated as physician or psychotherapist, because these professions are the main target group of the clinical guidelines. To reduce the dropout rate for BL, participants received information at project registration where the face-to-face workshops of the BL-condition were planned to take place (Berlin, Düsseldorf, Ulm). All persons who confirmed to be willing to attend a workshop in one of those cities, were included in the BL-condition. The other persons were randomly assigned to either the PEM- or EL-condition, with the PEM-condition being over randomized to account for the extended number of participants in the EL due to transfer from the BL- to the EL-condition (see ‘Participants and dropout’). Randomization was managed by an external company providing the website, system support and programming using computer-generated random numbers. Thus, the research team and participants were not aware of the randomization sequence; they were aware of the intervention assignment after allocation though. Participation in the trainings was free of charge, and participants were free to complete the training at their own pace as long as it was completed within three months.

The data were assessed pseudonymously via online questionnaires at three measuring points, T1-T3. Participants had to complete T1 to gain access to the training method to which they were assigned. After they completed the training, they were asked to fill in the post intervention questionnaire (T2). Participants received a certificate of training participation (including professional credits) only if they completed T2. As the surveys were conducted pseudonymously, the information and evaluation given by the participants in the questionnaire were not related to the certification. For certification, it was only important *that* T2 was completed, not *how* T2 was completed. Participants were informed of that procedure. Participants were regularly reminded by automatically sent emails to complete their training and assessments. Three months after the training was completed, they were asked to participate at T3. To encourage completion of T3, participants who had been assigned to the PEM-condition were offered the option to participate in the e-learning training after completing the T3-assessment, and participants who had been assigned to the EL- or BL-condition were offered the opportunity to receive the printed educational material after completing the questionnaire at T3. It took approximately 30 min to complete each assessment (T1, T2, T3).

The trial duration was adapted a priori according to the project funding. Four time points were defined when a cohort (consisting of the three study conditions) started and training participation was possible: 12/2018-02/2019; 04/2019-07/2019; 10/2019-01/2020; 09/2020-12/2020. The last cohort was postponed due to the COVID-19 pandemic in hopes of being able to conduct face-to-face workshops at a later date; ultimately, no in-person workshops could be held in the last cohort so that participants who were allocated to the BL-condition were transferred to the EL-condition. The study stopped once the follow-up assessments of the fourth cohort were completed.

### Sample size

Other studies exploring different approaches for the training of medical professionals in suicide prevention have shown large Cohen d effect sizes of approximately 1.0 for the acquisition of knowledge and between 0.7 and 1.1 for self-confidence [48, 50]. Additionally, a study among gatekeepers on the efficacy of a web-based adolescent suicide prevention programme revealed large effect sizes for actual knowledge, perceived knowledge and perceived self-confidence [51]. Given these findings, we decided to choose an estimated effect size of d = 0.7. To detect this effect size, assuming an alpha of 0.05 and a statistical power of 1 − beta = 0.90, the total sample size resulted in 131, i.e., 44 per group (calculations based on G-Power 3.1.9.7). As we observed dropout rates of approximately 60% from T1 to T3 in our own projects evaluating e-learning courses for professionals, we aimed for a minimum group size of *n* = 110 for T1 to allow for attrition over time.

### Ethical considerations

Participation in the study was voluntary, and the data were assessed pseudonymously. All participants were informed beforehand about the study and provided online informed consent. The online course included information on the national telephone helpline in case participants experienced emotional stress triggered by training content. This study was approved by the medical ethical committee of Ulm University (311/18) on 29th August 2018.

### Interventions

PEM consisted of an A5-size brochure with a short, compact summary of the NSSI guidelines structured in four topics: ‘Classification’, ‘Epidemiology and Aetiology’, ‘Diagnostic Assessment’ and ‘Intervention’ which included information on ‘Therapeutic Assessment’. Therapeutic assessment (TA) is a brief, manualized intervention based on cognitive-analytic therapy that can be delivered in different settings by professionals from a range of disciplines [60]. TA comprises three main elements: (1) construct a diagram with the individual vicious circle that includes triggering situations, dysfunctional basic assumptions, resulting behaviours and their consequences; (2) identify potential exits of the circle; and (3) subsequently, address an ‘understanding letter’ to the patient where the issues discussed during the session are summarized and a follow-up appointment for further therapeutic care is offered. TA training was shown to be feasible and was associated with improved quality of self-harm assessment [61]. In total, the PEM contained approximately 60 pages including a pocket card with a flow chart and key facts about NSSI. It was certified within the continuing medical education system with 2 CME points. Currently, the developed brochure is available on the project homepage [62].

Learning material for EL was provided as texts, interview clips with experts, case-based exercises, good practice videos and further information such as worksheet templates for therapy. The EL texts were nearly identical to the information given in PEM, but the additional material provided more in-depth insight into the topic. Compared to PEM, EL included a more descriptive module on ‘Therapeutic Assessment’ with a focus on exercising the application of TA (the processing time for the module was appx. 135 min). Therefore, first a good practice video and understanding letter were presented and afterwards participants were invited to perform the three main elements of TA (construction of a vicious cycle, identification of potential exits, writing of an understanding letter) on the platform based on a given case vignette with video sequences of a simulated therapist-patient TA session. The processing time of EL was estimated to be approximately 7.5 h. EL was accredited with 18 CME points. Currently, the online course is not available for participation.

The BL-condition was identical to the EL-condition, except for module 5 ‘Therapeutic Assessment’, which was taught during a half-day face-to-face workshop (appx. 3.5 h) instead of online, resulting in a total processing time of approximately 9 h. The face-to-face workshop was led by one to two members of the research team and of the STAR-consortium with clinical theoretical and/or practical expertise on NSSI (psychotherapists, psychiatrists). It was held in the last month of the three-month participation period and comprised a short summary of the learning contents of modules 1 to 4 (appx. 35 min) and a theoretical input to module 5, which included the same good practice video and understanding letter as in the EL-condition (appx. 80 min). Subsequently, participants were assigned to groups of two to complete an on-site role-play (patient-physician/psychotherapist) of TA (appx. 60 min). Afterwards, experiences and questions were discussed within the whole group (appx. 25 min). BL was also certified with 18 CME points.

### Outcome measures and evaluation of training quality

#### Primary outcomes: Competences and attitudes (assessed T1, T2, T3)

*Knowledge about NSSI* was measured with a self-administered multiple-choice test of 15 questions, which included five answer choices on average (range from 4 to 8). Two questions specified that exactly one choice is correct, and for further data analysis, one point was awarded for each question if it was answered correctly. The other 13 questions indicated that at least one of the choices was correct. For the data analysis, we checked for each choice whether it was answered correctly (i.e., wrong choices not ticked and true choices ticked), and points were awarded accordingly. This procedure led to possible results ranging from 0 points to 70 points. The Cronbach´s alpha in this study was 0.72, indicating an acceptable internal consistency. The questions and choices were created based on the content of the trainings. One example of a question is: ‘Which of the following statements about associated symptoms and comorbidities in NSSV is/are correct?’ with the following answer choices: ‘persons who injure themselves suffer from a borderline personality disorder’, ‘NSSI occurs only in combination with a mental disorder’, ‘children/adolescents who injure themselves are more likely to commit suicide in the future’, ‘people who injure themselves are suicidal’, ‘people who show self-injuring behaviour during adolescence are more likely to show destructive behaviour during adulthood, too (i.e. substance abuse)’, ‘affective disorders are among the most common comorbidities in NSSI’.

To assess *perceived competences regarding NSSI*, participants were asked to rate 10 items on a 5-point Likert scale from 1 (do not agree at all) to 5 (agree totally); for example ‘I know how to proceed in a case of NSSI’. These items were partly self-created and partly adapted from a questionnaire used in an evaluation of workshops about suicidality and self-injury for school staff [63]. For analyses, the mean of the sum score of all items was computed. The Cronbach´s alpha in this study was 0.89, indicating good internal consistency.

*Positive attitudes toward the effectiveness of NSSI treatment* were assessed with five items taken from an attitude scale developed by Crawford and colleagues [64] that included statements such as ‘It is not useful for children/adolescents who self-harms to have contact with me’. Participants could express their consent to the statements on a 4-point scale from 1 (do not agree at all) to 4 (agree totally). The mean of the sum score of all items was used for analyses. The Cronbach´s alpha in this study was 0.64, indicating a questionable internal consistency.

Participants were asked to rate their *attitudes toward NSSI and those who self-injure* on a 5-point Likert scale ranging from 1 (do not agree at all) to 5 (agree totally). The scale comprises 15 items, for example, ‘I find it hard to understand people who self-injure’. The scale was composed of self-created items and items from various other questionnaires and studies [31, 65–67]. Higher values reflect more negative attitudes toward NSSI and those who self-injure. The average sum score of all the items was used for the analyses. In this study, the newly composed scale reached a Cronbach´s alpha of 0.77, reflecting an acceptable internal consistency.

#### Transfer to practical work (assessed T3)

Only at T3 was the extent to which participants applied the short intervention ‘Therapeutic Assessment’, which was part of all trainings (yes /no/ not specified), assessed. If participants conducted TA, they were asked, how often (four categories from ‘up to ca. 25% cases of NSSI’ to ‘75 – 100%’) and which element(s) of TA was/were applied (construction of diagram, searching for potential exits, writing an understanding letter). Moreover, participants estimated how helpful they found application of TA on a 5-point Likert scale ranging from 1 (do not agree at all) to 5 (agree totally). If participants indicated not having applied TA, they could choose from a list of reasons why.

#### Training evaluation (T2)

To ensure the quality of the learning formats, user satisfaction and evaluation of their training were surveyed at T2 with 16–32 items, depending on the training condition. Nine items were evaluated in all three conditions, and seven items were rated on a 6-point scale ranging from 1 (not true at all) to 6 (absolutely true) (example: ‘the contents are relevant for my professional context’). Two items measured perceived level of training (5-point scale from 1 (too low) to 3 (exactly right) to 5 (too high)) and perceived depth of information (5-point scale from 1 (too superficial) to 3 (exactly right) to 5 (too specific)).

#### Demographic characteristics and covariates

The demographic characteristics collected included gender (male/female), year of birth, country of residence, profession (Medical Psychotherapist, Child and Adolescent Psychiatrist, Paediatrics, General Practitioner, Other Physician, Adult Psychotherapist, Child and Adolescent Psychotherapist) and working context (psychiatric, psychotherapeutic or psychosomatic clinic for children/adolescents; psychiatric, psychotherapeutic or psychosomatic clinic for adults; paediatric clinic; other clinic; psychiatric, psychotherapeutic or psychosomatic practice/outpatient clinic for children/adolescents; psychiatric, psychotherapeutic or psychosomatic practice/outpatient clinic for adults; paediatric practice/outpatient clinic, other practice/outpatient clinic; social paediatric centre; public health service; counselling work (e.g. specialist counselling centre, educational counselling, family counselling; other)). For description of demographic characteristics, working context was categorized into ‘inpatient’ (comprising the first four answer options listed above), ‘outpatient’ (comprising the following four answer options listed above) and ‘other’ (comprising the last four answer options listed above).

As covariates, the frequency of previous experience with NSSI in the work context, personal experience with people who self-injure (including one self) and attendance at previous trainings on the topic were surveyed at T1, as was attendance at other trainings on NSSI parallel to/after participation in the project at T2 and T3.

### Analyses

Statistical analyses were performed using the Statistical Package for the Social Sciences SPSS 28.0.1. For sample description, nominal data are presented as frequencies, while continuous data are presented as mean (M) and standard deviation (SD).

To analyse the overall difference between the three training conditions over time, linear mixed-effects models with fixed factors training (PEM, EL, BL), time (T1, T2, T3) and the interaction between the two were applied for the different primary outcome variables: knowledge about NSSI as assessed by the MC-Test, competences regarding NSSI, attitudes toward effectiveness of treatment and attitudes toward NSSI and those who self-injure. Graphical inspection of the normal distribution and homoscedasticity of the residuals of the dependent variables revealed no major violation of those assumptions. Furthermore, mixed-effects models have been shown to be robust against violations [68, 69].

The influence of covariates (age, sex, professional group (physician/psychotherapist), work experience, cases of NSSI confronted with professionally, personal experience with people who self-injure (including one self), and participation in other trainings on the topic during training participation) was analysed exploratorily by calculating mixed-effects models for each covariate separately, and significance of interaction effects, including the respective covariate (training x time x covariate), was checked.

As it was not possible to skip items in the online-questionnaires, there were no missing data within one questionnaire. Missing values within one proband between different measuring points due to dropout from the study were handled using the Full Information Maximum Likelihood (FIML) method which estimates model parameters by taking into account the available data under the assumption of missing-at-random which seems justified for the drop-outs.

Differences among trainings in practical transfer were analysed using one-way ANOVAs and chi-square tests. When appropriate, Cramer’s V (V) was calculated. The impact of covariates was explored by conducting a binary logistic analysis with the application of TA as a criterion and training approaches as well as the covariates listed above as predictors. For training evaluation, descriptive analyses and one-way ANOVAs were used. When conducting ANOVA, some variables violated the assumption of a normal distribution. As one-way ANOVA has proven to be robust against violations of a normal distribution [70, 71], it was still applied. When there was no homogeneity of variance, Welch ANOVA was conducted.

For all analyses, group assignments followed an, as-treated’ principle. For interpretation of the main analysis, i.e. the interaction effect of training x time for the four primary outcome variables, we corrected the level of significance from *p* = .05 to *p* = .0125 according to the test of four hypotheses. For interpretation of all the other analyses, a level of significance of *p* = .05 was applied, unless multiple comparisons between the three training conditions were conducted. In these cases, the critical p value was adjusted using Bonferroni adjustment.

## Results

### Participants and dropout

**Fig. 1:**
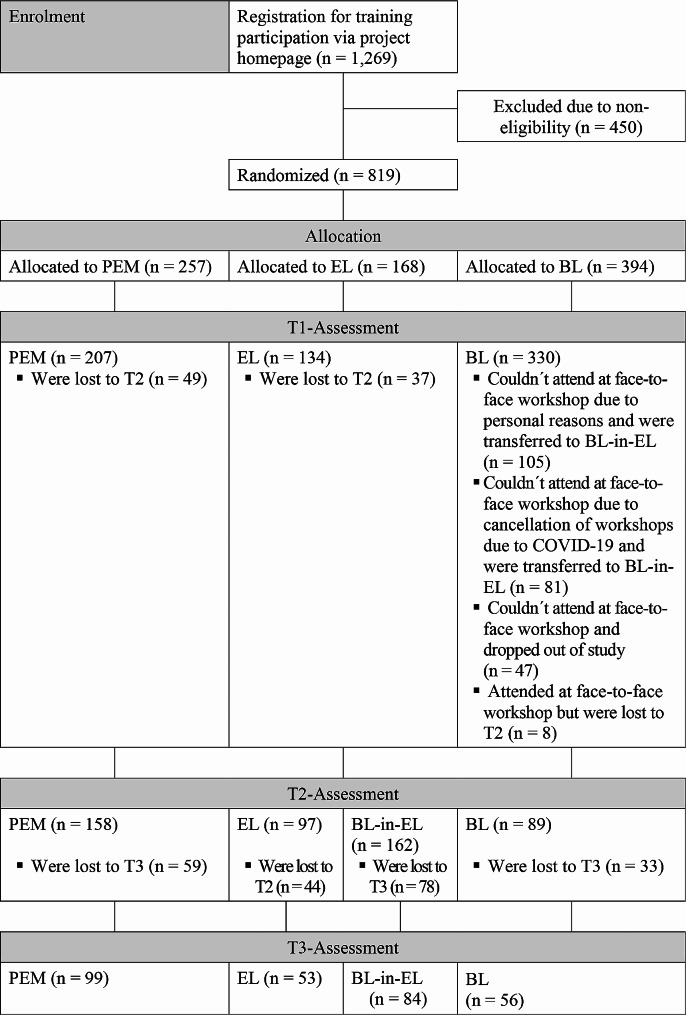
Flow of participants through the trial. PEM, printed material; EL, E-Learning; BL, Blended-Learning; BL-in-EL, participants originally allocated to BL who were transferred to EL due to nonparticipation at the workshop

In total, 1,269 persons registered on the project homepage to participate in one of the trainings. A total of 450 persons were excluded from the study because they did not live in Germany and/or did not indicate that they were physicians or psychotherapists. Of the remaining 819 persons, 257 were allocated to PEM, 168 to EL and 394 to BL. T1 assessment was completed by 207 participants (80.5%) in PEM, 134 participants (79.8%) in EL and 330 participants (83.8%) in BL. Participants assigned to BL who could not attend one of the offered face-to-face workshops (*n* = 186) were transferred to EL and thus were included in the EL sample. Participants who attended at one of the workshops and thus stayed in BL and completed T2 did not differ in age, gender, profession, work context, work experience or number of cases of NSSI being confronted with professionally, compared to participants who were transferred from BL to EL and completed T2 (‘BL-in-EL’), indicating that the randomization effect was not impaired by this procedure. This resulted in group sizes of PEM = 207, EL = 320 and BL = 144 for T1. Finally, outcome data were obtained for 158 participants in PEM, 259 in EL and 89 in BL (T2). Thus, of the 671 participants who completed T1 and had access to their training, 506 participants finished their training and completed T2 (dropout rate: 24.6%). The dropout rate from T1 to T2 was 23.7% in PEM, 27.6% in EL and 38.2% in BL (for EL and BL, the participants who were transferred from BL to EL were neglected because this would bias the dropout rate). No significant differences between those who dropped out and those participants who successfully completed the course and filled out T2 were found in terms of age, gender, profession, work context, work experience or number of cases of NSSI being confronted with professionally. We retained 99 participants (62.7%) of PEM, 137 (52.9%) for EL and 56 (62.9%) for BL for the 3-month follow-up assessment (T3). Therefore, in total 292 participants completed T3-assessment. There were no significant differences between the participants who dropped out from T2- to T3-assessment and the participants completing T3 for the listed variables. The flow of participants through the trial is illustrated in the Fig. 1. The sample characteristics of the dropout groups can be found in Additional file 1 (Table S1).

### Sample characteristics of participants who successfully completed their training

Demographic and work-related data of successful participants (i.e., participants who successfully completed all exams) are presented in Table 1. The majority of the sample was female, the average age was *M(SD)* = 43.80 (9.25) years. Most of the participants worked as psychotherapists. Slightly more worked in an outpatient context than in an inpatient context. On average, the participants had *M(SD)* = 10.34 (8.68) years of work experience.

**Table 1:** Sociodemographic characteristics of the participants at T2

Sociodemographic characteristic	Total sample		PEM		EL		BL	
	*n*	%	*n*	%	*n*	%	*n*	%
Gender								
Female	422	83.4	130	82.3	220	84.9	72	80.9
Male	84	16.6	28	17.7	39	15.1	17	19.1
Profession								
CAP	92	18.2	25	15.8	46	17.8	21	23.6
Paediatrics	31	6.1	8	5.1	19	7.3	4	4.5
Med PT	48	9.5	25	15.9	21	8.1	2	2.2
Other physician	9	1.8	3	1.9	4	1.5	2	2.2
APT	95	18.8	27	17.1	48	18.5	20	22.5
CAPT	231	45.7	70	44.3	121	46.7	40	44.9
Working context								
Inpatient	200	39.5	69	43.7	95	36.7	36	40.4
Outpatient	241	47.6	74	46.8	124	47.9	43	48.3
Other	65	12.8	15	9.5	40	15.4	10	11.2

Sociodemographic characteristic	Total sample		PEM		EL		BL	
	*M*	*SD*	*M*	*SD*	*M*	*SD*	*M*	*SD*
Age	43.80	9.25	42.34	9.01	44.11	9.41	45.48	8.92
Years of work experience	10.34	8.68	9.11	8.02	10.75	8.79	11.34	9.32

*N* = 506 for the total sample, *n* = 158 for PEM, *n* = 259 for EL, *n* = 89 for BL

PEM, printed material; EL, E-Learning; BL, Blended-Learning; CAP, Child and Adolescent Psychiatrist; Med PT, Medical Psychotherapist; APT, Adult Psychotherapist; CAPT, Child and Adolescent Psychotherapist

### Outcome data

#### Competences and attitudes

Table 2 depicts the estimated mean scores for the four dependent variables separated by the training method for each time point. Additional file 2 (Table S2) shows the descriptive statistics of the dependent variables by training condition and measurement point. To evaluate differences between the different training strategies over time, mixed effects models were calculated for each dependent variable including all time points and data from all 671 participants who completed T1 and started their training. All the dependent variables developed in the expected direction across the three measurement points (i.e., increase in mean values for knowledge about NSSI, competences regarding NSSI, positive attitudes toward the effectiveness of treatment and decrease in values for negative attitudes toward NSSI and those who self-injure). The statistical analyses revealed a significant main effect of time for all the variables (53.8 > *F* < 508.6, all ps < 0.001; not displayed in Table 2). Additionally, there was a significant training-time interaction effect for negative attitudes toward NSSI and those who self-injure. Comparisons of the estimated fixed parameters demonstrated that the effect of PEM differed significantly from that of EL and BL.

**Table 2:** Estimated means of dependent variables (emmeans) by training condition and measurement point

Variable	Group	T1		T2		T3		Analyses Group x Time
		*M*	*SE*	*M*	*SE*	*M*	*SE*	
Score (%) knowledge about NSSI	PEM	80.52	0.52	84.61	0.74	88.70	1.12	F (2, 1395) = 0.17, *p* = .844
	EL	79.97	0.42	84.34	0.59	88.71	0.90	
	BL	80.54	0.64	85.04	0.93	89.54	1.45	
	PEM vs. EL vs. BL	-		-		-		
Competences regarding NSSI	PEM	3.84	0.04	4.15	0.03	4.46	0.04	F (2, 1463) = 4.03, *p* = .018
	EL	3.75	0.03	4.16	0.03	4.56	0.03	
	BL	3.88	0.05	4.23	0.04	4.58	0.05	
	PEM vs. EL vs. BL	-		-		-		
Positive attitudes effectiveness of NSSI treatment	PEM	3.28	0.03	3.37	0.02	3.46	0.03	F (2, 1430) = 0.11, *p* = .896
	EL	3.33	0.02	3.42	0.02	3.51	0.03	
	BL	3.39	0.03	3.47	0.03	3.54	0.05	
	PEM vs. EL vs. BL	PEM < BL		-		-		
Negative attitudes NSSI and those who self-injure	PEM	2.04	0.03	1.92	0.03	1.81	0.03	F (2, 1463) = 5.54, *p* = .004
	EL	2.06	0.02	1.88	0.02	1.70	0.03	
	BL	2.06	0.04	1.87	0.03	1.68	0.04	
	PEM vs. EL vs. BL	-		-		PEM > EL		

The group assignments followed an, as-treated’ principle. Estimated mean values were calculated for each time point respectively. The last column ‘Analyses Group x Time’ shows the results of the training-time interaction of linear mixed-effect models including all time points and data from all 671 participants who completed T1. The critical level of significance was corrected from *p* = .05 to *p* = .0125 according to the testing of four hypotheses. In the line “PEM vs. EL vs. BL”, significant pairwise comparisons between training conditions based on the linear mixed model using emmeans and accounting for multiple comparisons (*p* < .05) are displayed

PEM, printed material; EL, E-Learning; BL, Blended-Learning

Explorative analyses of covariates revealed that only the interaction effect of training x time x sex was significant (*p* = .011) for the dependent variable ‘competences regarding NSSI’, indicating that male participants profited more from PEM over time than female participants and that female participants profited more from EL than male participants did (see Additional file 3 (Table S3) for more details). When multiple comparisons were accounted for by applying the Bonferroni adjustment, significance was not reached any more though.

#### Transfer to practical work

To compare the application rate of TA between training approaches, analyses were conducted while neglecting the answer option “not specified”. 48% of participants of BL-condition indicated having applied TA in their work setting (25 of 52) compared to 40% in EL (47 of 118) and 25% in PEM (17 of 69) (*χ*^*2*^ [2] = 7.64, *p* = .022, V = 0.179). To explore the impact of covariates on the application rate of TA, a binary logistic regression model was conducted with the application of TA as the criterion and the following predictors: dissemination strategy, age, sex, professional group, work experience, cases of NSSI confronted with professionally, personal experience with people who self-injure (including one self) and participation in other trainings on the topic. The model was statistically significant (χ² [10] = 23.59, *p* = .009, *N* = 238) and showed good model fit with a small amount of explained variance (Hosmer-Lemeshow-Test: χ² [8] = 9.01, *p* > .05; Nagelkerkes R² = 0.129). The significant predictors included dissemination strategy (*p* = .033, OR = 1.466, 95%-KI[1.032, 2.081], professional group (*p* = .002, OR = 0.334, 95%-KI[0.169, 0.659] and cases of NSSI confronted with professionally (*p* = .006, OR = 0.745, 95%-KI[0.603, 0.919] (see Additional file 4 (Table S4) for more details). Regarding the professional group, 22 of 79 physicians stated that they applied TA (27.8%), whereas 67 of 159 psychotherapists did (42.1%). Taking a closer look, physicians and psychotherapists did not differ in their application rate of TA in the EL-condition (16 of 41 physicans, 39%, vs. 31 of 76 psychotherapists, 40.8%), but in the BL-condition, 5 of 15 physicians (33.3%) applied TA, while 20 of 37 psychotherapists did (54.1%) and in the PEM-condition only 1 of 23 physicians applied TA (4.3%) compared to 16 of 46 psychotherapists (34.8%). With regard to the extent of professional contact with NSSI cases, our results indicate that the more often professionals have been confronted with NSSI cases, the more likely they were to apply TA.

Among those participants who applied TA, participants of PEM, EL and BL did not differ significantly in terms of the frequency of application. Among the three main elements of TA, ‘construction of diagram’ and ‘searching for potential exits’ were applied most often (appx. 90% respectively). Only a minority wrote an ‘understanding letter’. The training conditions did not differ in terms of the frequency of application. Estimation of how helpful participants found TA, if applied, was high and did not differ among trainings. Among the reasons for not applying TA, ‘necessary setting not present’ was among the most frequently mentioned reasons. Participants of BL had the lowest rates of reasons related to perceived deficits in competence or not feeling familiar enough with the intervention (see Table 3).

**Table 3:** Application of therapeutic assessment

Variable	PEM		EL		BL	
	*n*	*%*	*n*	*%*	*n*	*%*
Frequency of application of TA^b^						
Up to ~ 25% of cases of NSSI	3	17.6	15	31.9	11	44.0
25 − 50% of cases of NSSI	4	23.5	9	19.1	7	28.0
50 − 75% of cases of NSSI	6	35.3	16	34.0	6	24.0
75 − 100% of cases of NSSI	4	23.5	7	14.9	1	4.0
Applied parts of TA^a, c^						
Construction of diagram	15	88.2	42	89.4	22	88
Searching for potential exits	15	88.2	43	91.5	23	92
Writing understanding letter	4	23.5	3	6.4	4	16
Reasons for not applying TA^a, d^						
Wasn´t confronted with case of NSSI since course completion	14	26.9	15	21.1	6	21.4
Didn´t feel competent enough	3	5.8	9	12.7	0	0
Intervention not familiar enough	17	32.7	13	18.3	3	10.7
Not convinced of usefulness of intervention	4	7.7	1	1.4	0	0
Necessary setting not present	16	30.8	36	50.7	14	50.0
Other	7	13.5	7	9.9	5	17.9

Subsample sizes for ‘Frequency of application of TA’ and ‘Applied parts of TA’: *n* = 17 for PEM, *n* = 47 for EL, *n* = 25 for BL. Subsample sizes for ‘Reasons for not applying TA’: *n* = 52 for PEM, *n* = 71 for EL, *n* = 28 for BL. On a scale from 1 (do not agree at all) to 5 (agree totally), participants found the application of TA rather helpful on average with no significant differences between groups (*M(SD)* for PEM = 4.18 (0.39), *n* = 17, *M(SD)* for EL = 4.17 (0.52), *n* = 47, *M(SD)* for BL = 4.20 (0.41), *n* = 25; F [2] = 0.033, *p* = .967, f = 0.001)

TA, Therapeutic Assessment; PEM, printed material; EL, E-Learning; BL, Blended-Learning. The group assignments followed an, as-treated‘ principle

^a^Multiple answers possible

^b^χ^2^ [6] = 6.38, *p* = .382, V = 0.189

^c^χ^2^ [4] = 4.05, *p* = .399

^d^χ^2^ [10] = 23.19, *p* = .010. The results should be interpreted with caution because more than 20% of the cells had expected frequencies < 5 and because the cells had an expected frequency < 1

### Training evaluation

Table 4 shows agreement with different statements about quality and user satisfaction, which were assessed in all three conditions. Satisfaction with and evaluation of the three different training methods were positive, with most mean values being greater than 5 on a scale from 1 to 6 with 6 reflecting the most positive evaluation. In general, the results showed that participants in BL gave the best evaluation, while those in PEM gave the worst evaluations; moreover, some of those differences were significant.

**Table 4:** Evaluation of the different training conditions

Variable	PEM		EL		BL		Bonferroni post hoc
	*M*	*SD*	*M*	*SD*	*M*	*SD*	
The design of the website/ printed educational material is attractive.	5.09	0.84	4.87	0.96	5.12	0.77	n. sig.
The structure of the training is coherent.	5.35	0.69	5.42	0.78	5.48	0.59	n. sig.
The learning content is relevant for my professional activities.	5.25	0.81	5.40	0.83	5.53	0.66	n. sig.
The training method is an appropriate format for this issue.^a^	4.78	1.03	5.35	0.78	5.53	0.68	*p* < .001 for 1 vs. 2 and 1 vs. 3
In total, I´m satisfied with the training.^a^	5.16	0.85	5.43	0.71	5.64	0.56	*p* < .001 for 1 vs. 2 and 1 vs. 3
The expenditure of time I spent for the training was profitable.	5.27	0.91	5.42	0.82	5.52	0.73	n. sig.

The group assignments followed an, as-treated’ principle. The scale for all items was 1 (not true at all) – 6 (absolutely true). For Bonferroni post hoc analyses (last column), the critical p-value was adjusted to the three post hoc comparisons conducted, resulting in critical p-value = 0.017. PEM = 1, EL = 2 and BL = 3. *N* = 158 for PEM, *n* = 259 for EL, *n* = 89 for BL. n. sig. = not significant

PEM, printed material; EL, E-Learning; BL, Blended-Learning

^a^Due to unequal variances between groups, Welch ANOVA was conducted

The level of training was judged to be appropriate by 75.9% of PEM, 90% of EL and 89.9% of BL. The depth of information was perceived as being exactly right by 61.4% of PEM, 79.5% of EL and 82% of BL. A total of 38% of PEM stated to find the depth of information ‘a little too superficial’.

## Discussion

Given the high clinical relevance of NSSI, it is crucial to educate professionals effectively about evidence-based guideline recommendations. Our study showed that all three training formats developed for the dissemination of NSSI guidelines contributed to the intended changes in different variables, including an increase in knowledge about NSSI, an increase in perceived competences regarding NSSI and of self-evaluated positive attitudes toward the effectiveness of treatment, as well as a decrease in negative attitudes toward NSSI and those who self-injure. The training effect remained stable upon the follow-up assessment three months later. For all three dissemination strategies, user satisfaction was high and the evaluation of training quality was positive. We were able to attract a substantial number of physicians and psychotherapists to participate in the project. Thus, all three training formats seem to be of high quality and capable of covering the needs of a heterogeneous group of physicians and psychotherapists. Our hypotheses were partially confirmed. Comparing the different dissemination strategies for competences and attitudes, we found significant group x time interaction effects for negative attitudes; specifically, the PEM-condition was statistically significantly less effective than EL and BL were, but the clinical significance of the statistical differences must be judged as rather low and may be neglected. EL and BL did not significantly differ in these variables. Clear differences between training conditions were revealed in the transfer to practical work, with PEM having the lowest uptake rate of TA and BL the highest. Thus, H1 was not confirmed, but H2 was.

Therefore, our study suggested that all three training formats are beneficial for strengthening the competencies of physicians and psychotherapists in dealing with NSSI and achieve similar outcomes despite the very different conceptual didactical approaches of PEM compared to EL and BL, implying different levels of required human, financial and time resources for development and despite unequal processing time. The additional learning material incorporated in EL and BL (such as case-based exercises or videos) did not enhance the acquisition of knowledge, competences or positive attitudes in our study, contrary to our expectations. A meta-analysis by Richard et al. concluded that simulation (such as role-play) is promising for practical training in suicidal crisis intervention but they also stated that strength of the evidence is limited [55]. Greater differences in these variables between PEM and the other approaches might occur after a longer period of time. The good results of PEM may also reflect the benefits of a brief and compact educational approach by providing a summary of the guidelines for quick reference and the preference of (mental) health professionals for having something ‘on hand’ [72, 73]. Additionally, satisfaction with PEM was similar to EL- and BL-condition. One possible explanation for that finding is that participants felt that the effort required to complete their training was well proportionate to the perceived benefits. Moreover, participants knew that they would receive the opportunity to complete one of the other trainings (i.e. PEM received access to EL; EL and BL received PEM) once their condition was completed within the study. Thus, although it is the most passive dissemination strategy we included, our study suggested that well-developed print material can offer a well-accepted, inexpensive and feasible dissemination approach for guidelines [74].

In accordance with H2, BL had the highest application rate of TA. This could indicate that the possibility of practicing TA during the face-to-face workshop increases the chance that people actually apply TA in their work context. Also other findings have shown the potential of experiential exercises for the acquisition of clinical skills among health professionals in regard to suicidality and self-harm [49, 55, 61]. It should also be noted, however, that the three dissemination strategies differed not only in their training format but also in their processing time. Consequently, our findings could also imply that more extensive training is more effective for transferring to practical work. This finding is in line with the findings of the review by Frank, Becker-Haimes and Kendall on therapist training in evidence-based interventions for mental health, which revealed that more intensive training models show promise for changing therapist behaviour [53]. As former studies on TA training outcomes use more extensive training than our study does (e.g., five half-day TA training sessions [61]), it is encouraging that even less comprehensive TA training sessions, such as those used in our study, have positive effects. Our results indicate that physicians benefitted less than physicians from the PEM- and BL-training approaches regarding the application of TA. One explanation for that could be that, in EL, physicians were able to review material and good-practice videos as often as they needed, whereas psychotherapists could apply TA more quickly in PEM- and BL-condition because they are more familiar with the therapeutic nature of TA. If so, future dissemination strategies should tailor their content more to the respective target group. However, as these analyses were exploratory, the results should be interpreted with caution.

Taking the perspective of training providers, the study supports a dose-response relationship: the more investment is put into the development of a training, the more benefit it shows. Printed educational material is the training format with the lowest threshold for participation and it requires the least resources to develop compared to the other two training strategies – and had the least positive effects compared to one or both of the other training formats, although overall, it is still at a high level. Compared to PEM, an e-learning format requires more effort and resources to be developed. In our study, EL had better effects on some variables than did PEM. One might argue that those differences in efficacy are not enough to justify the extended efforts for the development of an e-learning training. On the other hand, an online course is easily scalable concerning the number of participants, once it is developed, and it is easier to update than PEM. Compared to EL, BL again needs a lot more resources for the organization and execution of the face-to-face part, especially considering the higher dropout rate we observed in the BL-condition. To reduce dropout, it would probably need more possible dates and venues for the face-to-face workshops and/or more flexibility are needed to retain participants in a BL format. For professionals, it is more challenging to integrate a face-to-face workshop in their daily work practice. In particular, the impact of the COVID-19 pandemic highlighted the need for more flexible training formats, such as online workshops. Another promising possibility would be to integrate a blended-learning format in existing infrastructures within an institution and use face-to-face workshops to practice newly acquired skills and/or case supervision [75, 76]. BL and EL did not differ in most of the variables assessed, but BL had the highest rate of TA application. Additionally, BL might support other desired outcomes (such as exchange, networking), we did not assess in comparison to the other two learning formats. If this justifies the greater effort of conducting BL compared to EL, it is surely a question of which training goals are aimed at being achieved. If the acquisition of practical capacity is the main focus, our study indicates that BL is the best option. Considering the positive effects we found for PEM, a combination of e-learning and printed educational material could be a good way to transfer competences and retain learning effects over time without causing financial and time barriers for both participants and providers, as a blended-learning format involves.

The subject of this study was the improvement of competences regarding and the application of clinical guidelines for NSSI in children and adolescents. It seems reasonable to assume that the results can also be transferred to the dissemination of other guidelines, although characteristics of the guidelines (level of evidence, length, practical orientation etc.) affect their actual use [73] and therefore might have an impact on the effect of different dissemination strategies. For the application of the Dutch multidisciplinary suicide prevention guideline, a blended-learning approach with an e-learning-supported Train-the-Trainer programme proved effective in improving guideline adherence, self-perceived knowledge and confidence of individual professionals working in psychiatric departments [48], as well as an e-learning module for undergraduate psychology students [50]. However, these studies did not compare different training formats. To our knowledge, there are no additional studies on the effectiveness of (different) dissemination strategies for other national guidelines for the management of NSSI or self-harm. Our results could inform future conceptualizations of guideline dissemination, for example, of the NICE guidelines on self-harm, especially given the fact that awareness and knowledge of the NICE guidelines were found to be low [27].

Generally, it is still unclear what makes the uptake of guideline recommendations into clinical practice successful. While some studies point toward the benefit of multifaceted interventions [21], more recent reviews have not found evidence that multifaceted interventions are more effective than single-component interventions [74, 77]. The competence of individual professionals may be only *one* part of a more comprehensive dissemination strategy in addition to implementation strategies targeting healthcare organizations and patients, suitable on-site structures of mental health care, acceptance of guidelines-based care by patients, or positive attitudes of professionals toward the use of guidelines [19, 78, 79], but it is undoubtedly a necessary part of any dissemination strategy. The positive effect of comprehensive or tailored implementation strategies has been demonstrated, for example, for the implementation of evidence-based practices for psychosis treatment [80], of guidelines for depression [78, 81] or of suicide prevention guidelines [82].

The strengths of the study include the direct comparison of different dissemination strategies with the inclusion of innovative training formats (e-learning and blended-learning), a large sample size and the multiple assessment design, including a 3-month follow-up. Limitations of the study include that the results might not be generalizable to other professionals, given that participants who enrolled in the project probably represent a self-selected sample of especially interested and motivated (mental) health professionals. Moreover, the participants had already good background knowledge about NSSI before starting the training which might impart the generalizability of this study, too, because it is still unclear whether prior knowledge has positive, negative or negligible effects on learning [83]. For example, on the one hand learners with high prior knowledge could show less gain of competences than learners with lower prior knowledge due to a ceiling effect or by preventing learners from finding new and better problem solutions than those they applied before. On the other hand, it is also possible that learners with high prior knowledge show a higher gain of competences than learners with lower prior knowledge because high prior knowledge facilitates the interpretation and encoding of new information [83]. Another limitation relates to the randomization procedure. Participants were partly randomized based on their preference (allocation to BL if attendance at workshop sites was judged as ‘possible’ beforehand). This can lead to a selective sample of more motivated participants in BL and biased estimates of treatment effects [84]. On the other hand, this step was necessary to enable the planning of the face-to-face workshops in advance (e.g., search for possible venue sites, decision on number of face-to-face workshops etc.) and the participation of interested healthcare professionals in the study, which, under the circumstance of full randomization, could not have enrolled. Comparative analyses showed that participants who attended one of the workshops and stayed in the BL-condition and participants who were transferred from the BL- to the EL-condition due to nonattendance at a workshop did not differ in demographic variables. This finding indicates that the randomization effect was not impaired by this procedure. Additionally, other studies including a face-to-face training condition on empirically supported treatment reported practical challenges in randomization, such that not all study participants could be randomized [54]. Sholomskas and colleagues argue that studies in which the ability to fully randomize participants is limited by differences in the practical demands associated with the various experimental conditions and conclusions regarding effectiveness based on non randomized groups may be similar to those based on randomized samples [54]. The dropout rate for BL was still fairly high, which limits the validity and reliability of the results of this group. On the other hand, providers might quite likely face similar barriers when offering face-to-face workshops. Dropout might even be greater in study conditions such as ours, especially when there is the opportunity to still receive training (in our case, EL) and no fees are charged. Another limitation of our study concerns the assessment methods used. Although we applied some items from validated questionnaires, we had to construct items ourselves because no existing validated measures were appropriate for our study purpose. Therefore, the final questionnaires were not validated in their used form. Even instruments for the clinical assessment of NSSI itself largely lack sufficient information on their validity and reliability [85]. Consequently, the observed improvements and evaluations might not be solely due to the dissemination format. Furthermore, as the questionnaires relied on self-reports, it should be noted that the effects found in our study do not prove that the trainings led to actual behavioural changes in participants in their clinical practice beyond self-reports and to a reduction in the severity of patients’ symptoms. Therefore, we cannot make statements about the clinical significance of our findings, but previous studies indicate that training professionals in guideline recommendations leads to improved patient care [19, 20]. Future research should focus on further comparisons of different training strategies (instead of comparing one training format to a (wait list) control condition). Particularly in light of the COVID-19 pandemic and its impact on further education approaches, formats of synchronous ‘face-to-face’ online workshops with focus on role-plays and exercises to foster practical capacities should be explored as possible replacements for non digital, ‘real-person’ workshops. Ideally, future studies should include outcome data objectively assessing the change in daily work practices of (mental) health care professionals and/or patient outcomes.

## Conclusions

There is a high prevalence of NSSI during childhood and adolescence, and NSSI can have negative long-term implications if left untreated. Effective training strategies are a crucial part of disseminating evidence-based clinical knowledge among (mental) health professionals and consequently of improving patient care. This is the first study comparing different training strategies for the dissemination of the consensus-based clinical guidelines for NSSI in childhood and adolescence. It shows that we developed high-quality, effective trainings on NSSI for (mental) health care professionals, going beyond the transfer of theoretical knowledge but also improving self-perceived (practical) competences, self-efficacy, and attitudes. The developed training strategies imply different levels of demanded resources for development and implementation, but they are all feasible and well-accepted means to disseminate the clinical guidelines. The results provide clues for the dissemination of other clinical guidelines in regard to training strategies for professionals. They indicate that the choice of training method could be driven by considering, which training goals are aimed at being achieved and the benefit-cost ratio allowing for tailored training approaches.

### Electronic supplementary material

Supplementary Material 1

Supplementary Material 2

Supplementary Material 3

Supplementary Material 4

## Data Availability

The datasets generated and/or analysed during the current study are not publicly available due to reasons of data security but are available from the corresponding author upon reasonable request.

## Abbreviations

ANOVA: Analysis of variance
appx: approximately
APT: Adult psychotherapist
BL: Blended-learning
CAP: Child and adolescent psychiatrist
CAPT: Child and adolescent psychotherapist
CME: Continuing medical education
COVID-19: Coronavirus disease 2019
DSM-5: Diagnostic and statistical manual of mental disorders, 5th version
e.g.: Exempli gratia = for example
EL: E-learning
etc.: et cetera = and other similar things
i.e.: Id est = that is
M: Mean
MC-Test: Multiple-choice-test
Med PT: Medical psychotherapist
n: Size of subsample
N: Size of total sample
NSSI: Non-suicidal self-injury
PEM: Printed educational materials
SD: Standard deviation
STAR: Self-injury: treatment, assessment, recovery
TA: Therapeutic assessment
