# Supplementary material for: Training approaches for the dissemination of clinical guidelines for NSSI: a quasi-experimental trial
Source: Child Adolesc Psychiatry Ment Health. 2024 Aug 10;18:99. doi: 10.1186/s13034-024-00789-x (PMC11317012; doi:10.1186/s13034-024-00789-x)
Supplement: Supplementary file 1 — Supplementary Material 1 [file 13034_2024_789_MOESM1_ESM.docx]

Table S1 Sociodemographic Characteristics of Participants Dropping Out Of The Study

| Sociodemographic characteristic | Dropout group from T1 to T2 (N = 165) | | Dropout group from T2 to T3 (N = 214) | |
| --- | --- | --- | --- | --- |
|  | *n* | *%* | *n* | *%* |
| Gender |  |  |  |  |
| Female | 132 | 80.0 | 182 | 85.0 |
| Male | 33 | 20.0 | 32 | 15.0 |
| Profession |  | |  | |
| CAP | 37 | 22.4 | 44 | 20.6 |
| Paediatrics | 3 | 1.8 | 13 | 6.1 |
| Med PT | 28 | 16.9 | 22 | 10.3 |
| Other physician | 3 | 1.8 | 3 | 1.4 |
| APT | 31 | 18.8 | 39 | 18.2 |
| CAPT | 63 | 38.2 | 93 | 43.5 |
| Working context |  | |  | |
| Inpatient | 70 | 42.4 | 80 | 37.4 |
| Outpatient | 79 | 47.9 | 103 | 48.1 |
| Other | 16 | 9.7 | 31 | 14.5 |
|  | *M* | *SD* | *M* | *SD* |
| Age | 43.92 | 10.20 | 43.29 | 9.39 |
| Years of work experience | 10.31 | 9.29 | 10.14 | 8.86 |

*Note.* PEM, printed material; EL, E-Learning; BL, Blended-Learning; CAP, Child and Adolescent Psychiatrist; Med PT, Medical Psychotherapist; APT, Adult Psychotherapist; CAPT, Child and Adolescent Psychotherapist.
