# Supplementary material for: Training approaches for the dissemination of clinical guidelines for NSSI: a quasi-experimental trial
Source: Child Adolesc Psychiatry Ment Health. 2024 Aug 10;18:99. doi: 10.1186/s13034-024-00789-x (PMC11317012; doi:10.1186/s13034-024-00789-x)
Supplement: Supplementary file 2 — Supplementary Material 2 [file 13034_2024_789_MOESM2_ESM.docx]

Table S2 Descriptive statistics of dependent variables by training condition and measurement point

| Variable | Group | T1 | | T2 | | T3 | |
| --- | --- | --- | --- | --- | --- | --- | --- |
|  |  | *M* | *SD* | *M* | *SD* | *M* | *SD* |
| Score (%) knowledge about NSSI | PEM | 79.11 | 7.60 | 90.04 | 5.07 | 85.80 | 6.00 |
|  | EL | 78.92 | 7.69 | 88.06 | 6.21 | 86.03 | 6.61 |
|  | BL | 79.67 | 7.71 | 89.02 | 5.62 | 85.94 | 7.43 |
| Competences regarding NSSI | PEM | 3.76 | .56 | 4.35 | .47 | 4.28 | .46 |
|  | EL | 3.68 | .64 | 4.34 | .43 | 4.39 | .40 |
|  | BL | 3.80 | .59 | 4.46 | .38 | 4.33 | .38 |
| Positive attitudes effectiveness of NSSI treatment | PEM | 3.26 | .38 | 3.42 | .38 | 3.41 | .39 |
|  | EL | 3.31 | .39 | 3.47 | .41 | 3.47 | .38 |
|  | BL | 3.36 | .35 | 3.53 | .35 | 3.45 | .34 |
| Negative attitudes NSSI and those who self-injure | PEM | 2.08 | .38 | 1.81 | .39 | 1.88 | .36 |
|  | EL | 2.10 | .44 | 1.78 | .39 | 1.81 | .39 |
|  | BL | 2.09 | .41 | 1.80 | .39 | 1.84 | .36 |

*Note.* PEM, printed material (n_T1_ = 207; n_T2_ = 158; n_T3_ = 99); EL, E-Learning (n_T1_ = 327; n_T2_ = 259; n_T3_ = 137); BL, Blended-Learning (n_T1_ = 137; n_T2_ = 89; n_T3_ = 56). The group assignments followed an ,as-treated‘ principle.
