# Supplementary material for: Training approaches for the dissemination of clinical guidelines for NSSI: a quasi-experimental trial
Source: Child Adolesc Psychiatry Ment Health. 2024 Aug 10;18:99. doi: 10.1186/s13034-024-00789-x (PMC11317012; doi:10.1186/s13034-024-00789-x)
Supplement: Supplementary file 3 — Supplementary Material 3 [file 13034_2024_789_MOESM3_ESM.docx]

Table S3 Explorative analyses of covariates

| Variable | Covariate | Analyses group x time x covariate |
| --- | --- | --- |
| SK | Age | F(3, 1389) = .925, p = .428 |
| SK | Work experience | F(3, 1389) = 1.364, p = .252 |
| SK | Sex | F(2, 1389) = .169, p = .844 |
| SK | Professional group | F(2, 1389) = .381, p = .683 |
| SK | Personal experience | F(2, 1389) = 1.332, p = .264 |
| SK | Participation at other trainings T1 | F(2, 1389) = .986, p = .374 |
| SK | Participation at other trainings T2 | F(2, 1223) = .009, p = .991 |
| SK | Participation at other trainings T3 | F(1, 825) = 1.325, p = .250 |
| SK | Cases of NSSI | F(12, 1359) = .956, p = .489 |
| C | Age | F(3, 1457) = 1.622, p = .182 |
| C | Work experience | F(3, 1457) = .678, p = .566 |
| C | Sex | F(2, 1457) = 4.490, p = .011 |
| C | Professional group | F(2, 1457) = .601, p = .548 |
| C | Personal experience | F(2, 1457) = 1.097, p = .334 |
| C | Participation at other trainings T1 | F(2, 1457) = 0.215, p = .807 |
| C | Participation at other trainings T2 | F(2, 1291) = 0.215, p = .807 |
| C | Participation at other trainings T3 | F(1, 865) = 3.835, p = .051 |
| C | Cases of NSSI | F(12, 1427) = 1.578, p = .207 |
| AE | Age | F(3, 1424) = 2.457, p = .061 |
| AE | Work experience | F(3, 1424) = 1.499, p = .213 |
| AE | Sex | F(2, 1424) = .359, p = .699 |
| AE | Professional group | F(2, 1424) = .254, p = .776 |
| AE | Personal experience | F(2, 1424) = .821, p = .440 |
| AE | Participation at other trainings T1 | F(2, 1424) = .199, p = .819 |
| AE | Participation at other trainings T2 | F(2, 1268) = .325, p = .722 |
| AE | Participation at other trainings T3 | F(1, 852) = .027, p = .871 |
| AE | Cases of NSSI | F(12, 1394) = .462, p = .937 |
| AN | Age | F(3, 1457) = 1.171, p = .320 |
| AN | Work experience | F(3, 1457) = .265, p = .851 |
| AN | Sex | F(2, 1457) = 1.499, p = .224 |
| AN | Professional group | F(2, 1457) = .566, p = .568 |
| AN | Personal experience | F(2, 1457) = .445, p = .641 |
| AN | Participation at other trainings T1 | F(2, 1457) = .134, p = .875 |
| AN | Participation at other trainings T2 | F(2, 1291) = .569, p = .566 |
| AN | Participation at other trainings T3 | F(1, 865) = .077, p = .781 |
| AN | Cases of NSSI | F(12, 1427) = 1.394, p = .162 |

*Note.* SK, Score (%) knowledge about NSSI; C, Competences; AE, Positive attitudes toward effectiveness of NSSI treatment; AN, Negative attitudes toward NSSI and those who self-injure. Continuous covariates: age and work experience (years); binary covariates: sex (male/female), professional group (physician/psychotherapist), personal experience with people who self-injure (yes/no), participation at other training on the topic (yes/no); categorial covariates: cases of NSSI confronted with professionally (no/yes, with 1-3 cases/yes, with 4-10 cases/yes, with 11-20 cases/yes, with 21-50 cases/yes, with 51-100 cases/yes with > 100 cases). The influence of the covariates was analysed exploratorily by calculating mixed-effects models for each covariate separately and significance of interaction effects including the respective covariate (training (PEM = printed material/ EL = E-Learning/ BL = Blended-Learning) x time (T1, T2, T3) x covariate) was checked.
