# Supplementary material for: Training approaches for the dissemination of clinical guidelines for NSSI: a quasi-experimental trial
Source: Child Adolesc Psychiatry Ment Health. 2024 Aug 10;18:99. doi: 10.1186/s13034-024-00789-x (PMC11317012; doi:10.1186/s13034-024-00789-x)
Supplement: Supplementary file 4 — Supplementary Material 4 [file 13034_2024_789_MOESM4_ESM.docx]

Table S4 Application rate of TA predicted by dissemination strategy, age, sex, professional group, work experience, cases of NSSI confronted with professionally, personal experience with people who self-injure and participation at other trainings on the topic (N = 238)

|  | B | SE | Wald | p | OR | 95% CI for Odds Ratio | |
| --- | --- | --- | --- | --- | --- | --- | --- |
|  |  |  |  |  |  | Lower Bound | Upper Bound |
| Dissemination strategy | .382* | .179 | 4.564 | .033 | 1.466 | 1.032 | 2.081 |
| Age | -.039 | .023 | 2.876 | .090 | .962 | .920 | 1.006 |
| Work experience | .008 | .024 | .116 | .734 | 1.008 | .962 | 1.056 |
| Sex | .389 | .391 | .9990 | .320 | 1.476 | .686 | 3.179 |
| Professional group | -1.096** | .347 | 9.996 | .002 | .334 | .169 | .659 |
| Personal experience | .294 | .302 | .946 | .331 | 1.341 | .742 | 2.424 |
| Participation at other trainings T1 | .037 | .370 | .010 | .920 | 1.038 | .503 | 2.143 |
| Participation at other trainings T2 | .253 | .738 | .118 | .731 | 1.288 | .304 | 5.467 |
| Participation at other trainings T3 | .088 | .906 | .009 | .923 | 1.092 | .185 | 6.453 |
| Cases of NSSI | -.295** | .107 | 7.571 | .006 | .745 | .603 | .919 |
| Constant | 2.791 | 2.507 | 1.239 | .266 | 16.303 |  |  |

*Note.* TA, therapeutic assessment. Degrees of freedom were 1 for all Wald statistics. Codes for sex: 1 = female, 2 = male. Codes for professional group: 1 = physician, 2 = psychotherapist. Codes for personal experience: 1 = yes, 2 = no. Codes for participation at other trainings: 1 = yes, 2 = no.
